# A smartphone application to facilitate adherence to home-based exercise after flexor tendon repair: A randomised controlled trial

**DOI:** 10.1177/0269215520962287

**Published:** 2020-10-11

**Authors:** Jonas Svingen, Jenny Rosengren, Christina Turesson, Marianne Arner

**Affiliations:** 1 Department of Clinical Science and Education, Karolinska Institutet, Södersjukhuset, Stockholm, Sweden; 2 Department of Hand Surgery, Södersjukhuset, Stockholm, Sweden; 3 Department of Hand Surgery, Plastic Surgery and Burns, and Department of Biomedical and Clinical Sciences Experimental, Linköping University, Linkoping, Sweden; 4 Department of Health, Medicine and Caring Sciences, Linköping University, Linkoping, Sweden

**Keywords:** Smartphone, rehabilitation, adherence, hand therapy, flexor tendon

## Abstract

**Objective:**

Evaluate the effect of a smartphone application on exercise adherence, range of motion and self-efficacy compared to standard rehabilitation after repair of the flexor digitorum profundus tendon.

**Design:**

Prospective multi-centre randomised controlled trial.

**Setting:**

Four hand surgery departments in Sweden.

**Subjects:**

A total of 101 patients (35 women) (mean age 37.5 ± 12.8) were randomised to control (*n* = 49) or intervention group (*n* = 52).

**Intervention:**

A smartphone application to facilitate rehabilitation.

**Main outcome measures:**

Adherence assessed with the Sport Injury Rehabilitation Adherence Scale at two and six weeks (primary outcome). Secondary outcomes were self-reported adherence in three domains assessed at two and six weeks, self-efficacy assessed with Athlete Injury Self-Efficacy Questionnaire at baseline, two and six weeks. Range of motion and perceived satisfaction with rehabilitation and information were assessed at 12 weeks.

**Results:**

Twenty-five patients were lost to follow-up. There was no significant between group difference in Sport Injury Rehabilitation Adherence Scale at two or six weeks, mean scores (confidence interval, CI 95%) 12.5 (CI 11.8–13.3), 11.8 (CI 11.0–12.8) for the intervention group, and 13.3 (CI 12.6–14.0), 12.8 (CI 12.0–13.7) for the control group. Self-reported adherence for exercise frequency at six weeks was significantly better for the intervention group, 93.2 (CI 86.9–99.5) compared to the controls 82.9 (CI 76.9–88.8) (*P* = 0.02). There were no differences in range of motion, self-efficacy or satisfaction.

**Conclusion:**

The smartphone application used in this study did not improve adherence, self-efficacy or range of motion compared to standard rehabilitation for flexor tendon injuries. Further research regarding smartphone applications is needed.

**Level of evidence:**

I. Randomised controlled trial

Tendon rupture and adhesions are common problems during the rehabilitation of patients with flexor tendon injuries in the hand and reoperation rates of up to 13% have been reported.^1^ Home exercise programmes are considered important for reaching a successful outcome after flexor tendon repair.^2,3^ In these exercise programmes there is a delicate balance between getting enough tendon motion to minimise adhesions and having a load that is low enough to prevent tendon rupture. These programmes consist of regular home-based exercises, often recommended by a physiotherapist or an occupational therapist to do on an hourly basis in order to prevent joint stiffness and adhesions. This creates high demands on patients and their adherence to the rehabilitation protocol. Poor adherence to restrictions has been linked to an increased risk of tendon rupture after flexor tendon repair.^4,5^

Patients belief in their own ability (self-efficacy) to perform the necessary exercises has been shown to predict adherence to home-based physical therapy in general.^6^ However, the lack of studies on adherence to home based exercise after flexor tendon repair makes the evidence on how to improve adherence insufficient and the impact on clinical outcome unclear in this patient group.^7,8^

It has been shown that a smartphone application can be an effective tool for increasing adherence to home-based exercise.^9,10^ However, evidence that the intervention improves exercise adherence after traumatic conditions in the upper limbs is still insufficient.^8^ The main aim of this study was to explore a new and specifically designed smartphone application for flexor tendon rehabilitation and the effect on adherence to home-based exercise, self-efficacy and finger range of motion. We hypothesised that, compared to the control group, the intervention group that received the smartphone application would show a significantly higher adherence to exercise and self-efficacy after two and six weeks of rehabilitation and a higher total active finger range of motion at 12 weeks after surgery.

## Methods

Patients were recruited to this prospective multi-center randomised controlled trial at four specialised hand surgery units in Sweden (Stockholm, Uppsala, Örebro, and Malmö) starting in March 2017, with the last follow-up in May 2019. The study was registered at ClinicalTrials.gov (identification number NCT03812978). The Helsinki declaration was followed. Ethics were approved by the Regional Ethics Committee in Stockholm, Sweden. (Dnr 2016/2489-31/2). Written confirmed consent was obtained from all participants. Organisation responsible for conducting the study was the Department of hand surgery at Södersjukhuset, Stockholm, Sweden. The development of the smartphone application was funded by the Swedish national quality registry for hand surgery, HAKIR.

Patients who had been operated with a repair of a complete laceration of the flexor digitorum profundus tendon in a finger, with or without injury to the flexor digitorum superficialis tendon, were included in the study. Patients were only invited to participate if they had been assessed as suited for an early active motion protocol, were over 18 years old, owned a smartphone, spoke Swedish fluently, and were willing to participate in the study. Criteria for exclusion were concomitant fracture, or injury to the flexor pollicis longus or an extensor tendon. Due to a slow inclusion rate, the inclusion criteria were expanded in March 2018 to also include patients with injury to more than one finger.

Participants were randomised via a computer-generated concealed block randomisation method with envelopes to the intervention and the control group. The envelopes held a computer-generated allocation number for either the intervention or the control group. A total of 140 envelopes were created. The envelopes were sealed and held separately until a participant had passed the check concerning the inclusion and exclusion criteria. The envelope was then opened to reveal to which group the participant had been allocated. This process was performed by the treating physiotherapist at the end of the first postoperative appointment. No blinding of the participants or the outcome accessors was done as it was practically impossible in the clinical setting.

Both groups received standard hand rehabilitation for flexor tendon repair which was initiated within the first seven days by a physiotherapist. The standard rehabilitation protocol consisted of instructions on home exercises, information about the injury, restrictions regarding daily use of the hands in activities. Participants were recommended not to use their injured hand during the first four weeks. The home exercise programme consisted of early active motion exercises within a dorsal cast or splint during the first four weeks and included passive flexion, active flexion with gradually increased motion, and extension of the interphalangeal joints. After the cast had been removed, the range of motion and intensity of the rehabilitation was gradually increased according to protocol in the same way for both groups. The number of exercise sessions per day and number of repetitions was recommended after assessment of the participants’ individual needs but ranged from every fourth hour to hourly and with 3 to 10 repetitions. These assessments were done during appointments to the physiotherapist which were planned five times during the rehabilitation period of 12 weeks, altered according to the participant’s need. The instructions during appointments were given orally and as written information.

The intervention group received standard rehabilitation plus a smartphone application called ‘Böjsenskada’ (flexor tendon injury). The application was developed in a collaboration between the research group and the company AppInMed (AppInMed AB, Lund, Sweden. No grants were given). The application is available in Appstore and Google play (password for download 11883). The password for downloading the application was only given to participants in the intervention group in order to avoid access for the control group. The application was designed to improve the participants adherence to home-based exercise partly by including methods to invigorate self-efficacy. The application included a video on the three exercises for early active motion, push-notifications for exercise that were set at the prescribed exercise intensity, an exercise diary in a calendar view, written information about the surgery, anatomy, rehabilitation, restrictions on how to use the injured hand, questions and answers. A written and oral presentation of the features of the app was presented when a participant was allocated to the intervention group at the first visit to the physiotherapist. The participants were then recommended to use the app as they wanted. If needed, support for the settings of the app was given during ordinary appointments with the physiotherapist.

The treating physiotherapist completed the Sport Injury Rehabilitation Adherence Scale^11,12^ (Supplemental Appendix 1) at the follow-up at two and six weeks after surgery. The phrase ‘since the last appointment’ was used instead of ‘during today’s appointment’ as suggested by Brewer et al.^11^ The Sport Injury Rehabilitation Adherence Scale has demonstrated good reliability and validity.^11,13^ The score was calculated as a total of the three items in the test, ranging from 3 to 15 points, 15 indicating maximum adherence.

Self-reported adherence was assessed with a questionnaire divided into three domains as described by Milne et al.^14^ and Wesch et al.:^15^ frequency, duration and quality. The participants completed the questionnaire at visits to the physiotherapist at two and six weeks after surgery. To assess the domains of frequency and duration, participants answered four questions about completed and recommended frequency and duration of exercise. The response options were modified to fit home-based exercise after flexor tendon surgery (English version Supplemental Appendix 2, Swedish version Supplemental Appendix 3). A percentage of adherence was calculated by dividing the answers of the questions in each domain; completed exercises performed by the participant divided by prescribed exercises by the physiotherapist. Zero indicates no adherence and 100, complete adherence. The domain of perceived exercise quality in general was assessed using one question were the participants stated percentage of time when they had experienced good quality exercise (English version Supplemental Appendix 4, Swedish version Supplemental Appendix 5). The questionnaire has not previously been tested for validity or reliability.

In order to evaluate the participants’ changes in self-efficacy during the rehabilitation period, they completed a Swedish translation of the Athletic Injury Self-Efficacy Questionnaire^16^ at the first visit, and at two and six weeks after surgery (Swedish version Supplemental Appendix 6). The questionnaire was translated from English to Swedish and then back to English again to ensure a good translation. This was done by a bilingual person. The Athletic Injury Self-Efficacy Question-naire has been shown to have sound psychometric properties,^14,16^ but the Swedish version has not been tested for validity or reliability. The questionnaire consists of ten questions. Each question was scored ranging from 0% to 100%, zero percent indicating no agreement with the stated question, and 100 indicating full agreement. The mean score of the ten questions was calculated.

Range of motion was measured by the treating physiotherapist using a finger goniometer according to the manual of the Swedish quality registry for hand surgery (HAKIR; www.HAKIR.se). Isolated active joint motion (flexion and extension) of the proximal interphalangeal joint and distal interphalangeal joint was measured during full fist position and full extension at 12 weeks after surgery. Total active motion for the proximal interphalangeal joint and distal interphalangeal joint was then calculated. In patients with multiple finger injuries, a mean value from injured fingers was calculated.

To assess perceived satisfaction, participants were asked two questions: ‘How do you perceive the rehabilitation after your operation?’ and ‘How do you perceive the information after your operation?’ Answers were given on a visual analogue scale ranging from zero (no satisfaction) to 100 (maximum satisfaction). The intervention group also answered two questions about usage of the application on a visual analogue scale: ‘How often have you used the smartphone application?’ ranging from never to always, and ‘How helpful has the application been?’ ranging from not helpful to very helpful.

Sample size was calculated to test the two null hypotheses that the mean values of the Sport Injury Rehabilitation Adherence Scale score and total active motion, would be equal for the two groups. The criterion for significance was set at 0.05. The tests were two-tailed. To reach a power of 80% the sample size had to be 12 participants for the Sport Injury Rehabilitation Adherence Scale score and 57 for total active motion for each group. We assumed that the common within group standard deviation should be 2.5 and the mean difference should be 3 for adherence. For total active motion we assumed a within group standard deviation of 37.6 and a mean difference of 20. To compensate for a dropout rate of around 20% we planned to include 140 patients in the study.

Data was analysed with SPSS statistical software version 26 (IBM) using an intention-to-treat analysis including all randomised data. We compared group demographics with a chi-square test (injured hand, gender, injured digits, injured tendons and digital nerves) and an independent sample *t*-test (age). In order to answer the hypotheses, we used a mixed model to test the effect of the intervention and time. Three different covariance structures were tested on each outcome variable: unstructured, first-order autoregressive and compound symmetry. The unstructured covariance structure was the best model for all variables based on the model testing done. We chose this method because it is preferable when you have repeated measures and missing data.^17^ To test the effect of the two different groups at each separate time point, an interaction effect between time and groups was tested in the selected model.

## Results

**Figure 1.**
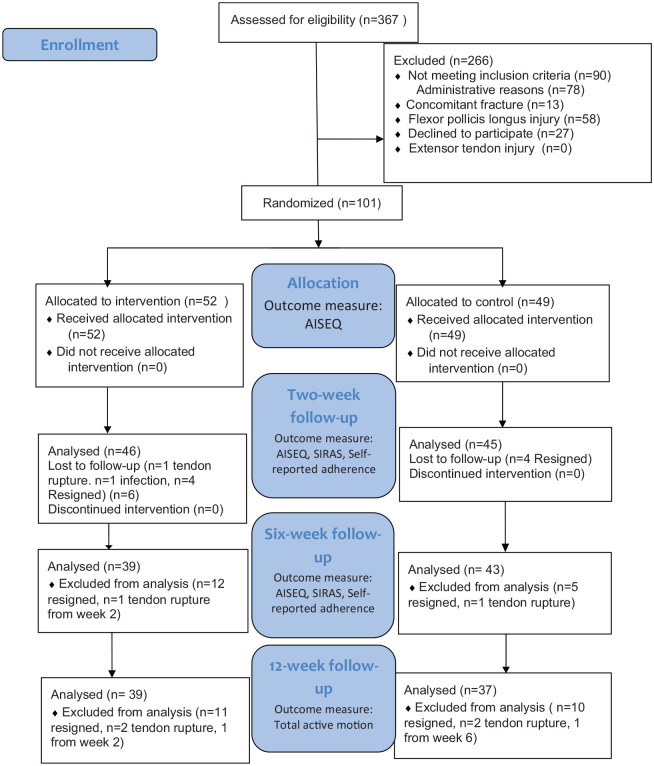
CONSORT flowchart of the study. SIRAS: Sport Injury Rehabilitation Adherence Scale; AISEQ: Athletic Injury Self-Efficacy Questionnaire. Total active motion: range of motion in the proximal and distal interphalangeal joints.

Figure 1 shows the numbers of participants in the different stages of the trial. Due to a slow inclusion rate we stopped the inclusion after inclusion of 101 participants. Several participants were excluded for administrative reasons; this was mainly due to sick leave among the physiotherapists planned to do the inclusions.

No statistical differences were found in demographics between the groups (Table 1). 35 women and 66 men with a mean age of 37.5 (SD 12.8) were randomised to intervention (*n* = 52) or control (*n* = 49). Six participants in the intervention group and four participants in the control group had an injury to more than one finger.

**Table 1.** Demographics of the participants in the intervention and control group.

Demographic	Intervention group (*n* = 52)	Control group (*n* = 49)	*P* value
Age (mean ± SD)	38.8 ± 13.4	36.2 ± 12.1	0.283
Gender (women/men)	21/31 (40/60%)	14/35 (29/71%)	0.212
Injured hand (Right/left)	24/28 (46/54%)	25/24 (51/49%	0.553
Injured digits, *n*	60	53	0.292
Index	24 (40%)	12 (23%)	
Middle	10 (17%)	6 (11.5%)	
Ring	9 (15%)	7 (13.5%)	
Small	17 (28%)	28 (52%)	
Injured tendons, *n*	81	83	0.661
FDP	31 (56%)	29 (52%)	
FDP + partial FDS	6 (10%)	8 (14%)	
FDP + FDS	19 (34%)	19 (34%)	
Injured digital nerves, n	30	22	0.444
None	36 (60%)	34 (64%)	
Unilateral	18 (30%)	16 (30%)	
Bilateral	6 (10%)	3 (6 %)	

FDP: flexor digitorum profundus; FDS: flexor digitorum superficialis.

Data is presented as mean (m), standard deviation (SD), number of participants (n) and proportion (%).

Six participants in the intervention group and four participants in the control group had an injury to more than one finger.

There were five serious complications: one infection in the intervention group, four tendon ruptures, two in the intervention and two in the control group. The complications were not judged to be related to the study since the rupture rates (3.8% and 4.1%, respectively) were within ordinary limits for this patient group.

There was no significant difference in total active range of motion in the injured finger between groups at 12 weeks after surgery (*P* = 0.18). Mean active range of motion was 95° for the intervention group and 108° for the control group (Table 2).

**Table 2.** Scores on all outcome measures at different time points for intervention and control groups.

Outcome variable	Follow-up	Intervention group	Control group	*P*-value
		Mean (95% CI)	Mean (95% CI)	
ROM*	Baseline			
	2 weeks			
	6 weeks			
	12 weeks	95 (83–108)	108 (94–123)	0.180
SIRAS	Baseline			
	2 weeks	12.5 (11.8–13.3)	13.3 (12.6–14.0)	0.155
	6 weeks	11.8 (11.0–12.8)	12.8 (12.0–13.7)	0.123
	12 weeks			
SR adherence for exercise frequency	Baseline			
	2 weeks	94.3 (90.7–98.0)	94.6 (90.8–98.3)	0.933
	6 weeks	93.2 (86.9–99.5)	82.9 (76.9–88.8)	**0.020**
	12 weeks			
SR adherence for exercise duration	Baseline			
	2 weeks	94.9 (90.6–99.3)	93.3 ± 16 (88.9–97.6)	0.586
	6 weeks	93.8 (89.1–98.5)	92.6 (88.2–97.0)	0.721
	12 weeks			
SR adherence for exercise quality	Baseline			
	2 weeks	86.7 (83.3–90.1)	87.1 (83.8–90.5)	0.866
	6 weeks	87.6 (83.4–91.9)	82.5 (78.4–86.7)	0.092
	12 weeks			
AISEQ	Baseline	91.5 (88.8–94.2)	90.5 (87.6–93.7)	0.994
	2 weeks	92.8 (90.7–94.9)	92.9 (90.2–95.6)	0.614
	6 weeks	91.5 (89.3–93.8)	90.4 (87.4–93.5)	0.600
	12 weeks			

ROM: range of motion in the proximal interphalangeal and distal interphalangeal joint; SR: self-reported; SIRAS: Sport Injury Rehabilitation Adherence Scale; AISEQ: Athletic Injury Self-Efficacy Questionnaire. Significant difference between the groups is in bold.

* ROM outcomes assessed only at 12 weeks.

There was no overall significant difference between the groups in Sport Injury Rehabilitation Adherence Scale (*P* = 0.096) or self-reported adherence in the domains of duration (*P* = 0.532), quality (*P* = 0.303) or frequency (*P* = 0.366) when tested for repeated measures with mixed model unstructured covariance structure. At two and six weeks the mean scores for Sport injury rehabilitation adherence scale were 12.5 (CI 95% 11.8–13.3) and 11.8 (CI 95% 11.0–12.8) respectively, for the intervention group and 13.3 (CI 95% 12.6–14.0) and 12.8 (CI 95% 12.0–13.7) for the control group. When testing the difference between groups at different time points for any adherence outcome measure the only significant difference was at six weeks for self-reported adherence for exercise frequency, with a higher mean score in the intervention group 93.2 (CI 95% 86.9–99.5) compared to the control group 82.9 (CI 95% 76.9–88.8) (*P* = 0.02) (Table 2).

There was a significant overall effect of time, independent of group, for self-reported adherence for exercise frequency (*P* = 0.007) with a higher reported adherence at two weeks compared to six weeks. There was no significant effect of time for Sport Injury Rehabilitation Adherence Scale and self-reported adherence for the domain of quality and duration.

Baseline scores for the Athletic Injury Self-Efficacy Questionnaire did not differ between the groups (*P* = 0.892). A mixed model with unstructured covariance structure showed no significant difference between groups over time (*P* = 0.632), but a significant effect of time independent of group (*P* = 0.008) with a higher value at two weeks compared with baseline and six weeks.

There was no significant difference between the groups in perceived satisfaction with rehabilitation and information (Table 3). In the intervention group, the mean score for the question ‘How often have you used the smartphone application?’ and ‘How helpful has the application been?’ was 49 for both questions.

**Table 3.** Results of the questions about perceived satisfaction with rehabilitation and information, and frequency of using the Smartphone application (SPA), and perceived helpfulness of the smartphone application at 12 weeks.

	Intervention group mean (95% CI)	Control group mean (95% CI)	*P* value
Perceived rehabilitation	93 (90.2–96.5))	93 (90.2–97.1)	0.645
Perceived information	92.5 (88.6–96.4)	95 (91.4–98.6)	0.332
Frequency of SPA	49 (38.3–60)	N/A	
Helpfulness of SPA	49 (37.2–61.1)	N/A	

## Discussion

The study showed that there was no overall significant difference between the intervention group and controls regarding adherence, self-efficacy, or total active motion. However, there was a significant difference in self-reported adherence for exercise frequency at six weeks follow up with a higher score in the intervention group. This difference was very small and maybe due to the loss of participants between two and six weeks. The results of the present study imply that the smartphone application in its present form failed to enhance adherence to the rehabilitation program among the participants.

Lambert et al. found that a smartphone application can improve self-reported adherence for home exercise.^9^ However, their intervention included motivational text messages and follow-up phone calls. Text messages alone have also been shown to increase medical adherence, especially if the text is personal.^18^ Like the reminding function of a text message or phone call, we used pop-ups in the smartphone application as reminders of exercise, but these pop-ups had the same text each time and were therefore not personal to the user. This may be one explanation to the lack of difference between the groups. Smartphone applications with instructional feedback have previously been shown to be able to reduce errors during exercise,^19^ and the lack of instructional feedback on exercise performance was a limitation of our smartphone application that may have affected the quality of exercise. Future improvements to smartphone applications for flexor tendon rehabilitations should probably include some type of personal feedback on exercise adherence for the user, both on quality and frequency of exercise.

In our study, the smartphone application also failed to improve the clinical outcome in participants, measured as finger range of motion. This result is similar to previous studies using smartphone applications.^10,20^ Total active range of motion results in the present study were similar to the study by Wiig et al.^21^ The total active motion was 95° in the intervention group and 108° in the control group and 35% of the patients were rated as Good or Excellent according to the Strickland original classification.^22^

A major limitation in our study was the high number of participants lost to follow up (25%) at week 12. This together with the insufficient inclusion of participants made it harder to answer our research question.

Another limitation of our study was that the participants were not blinded to the allocation groups as the control group could not receive a placebo smartphone application. The assessors of Sport Injury Rehabilitation Adherence Scale and range of motion were also not blinded to the allocation due to practical reasons concerning the clinical setting of the study. There is a lack of golden standards in evaluating adherence to home-based exercise in hand rehabilitation.^23^ The Sport Injury Rehabilitation Adherence Scale has previously been used to assess adherence to hand therapy,^24^ however, it has not been validated for patients with flexor tendon injuries and the phrase ‘since the last appointment’ was used instead of ‘today’s appointment’ which may compromise previously established validity and reliability.

Chen et al. reported a significant difference between self-reported exercise adherence when comparing the patient’s memory to information in the medical charts.^25^ Patients tended to overestimate their adherence. This may suggest that our high self-reported adherence rates of 80% to 100% may be overestimated. In order to test the first hypothesis that the intervention would increase adherence. We used two different methods as suggested by the World Health Organization:^26^ self-reported adherence by participants, as well as physiotherapist-rated adherence.

The participants in our study reported a high mean Athletic Injury Self-Efficacy Questionnaire score, suggesting a high self-efficacy for the prescribed exercise. Self-efficacy for home-based exercise has previously been shown to predict adherence,^14^ which may explain the high self-reported adherence in the present study.

All outcome measures declined between two and six weeks, and this effect of time was significant both for self-reported adherence for exercise frequency and Athletic Injury Self-Efficacy Questionnaire scores. The prescribed frequency and duration of exercise was higher at six weeks, inducing a greater effort for exercise in the patients which possibly affected the adherence rates. Huang et al.^27^ found a decline in exercise rates over time and an association to the perceived importance of exercise. As hand function improves over time, the perceived importance of exercise may decline, since the injury is perceived as less severe. This probably affects the motivation for exercise and may explain some of the loss in adherence over time. We believe that this also should be considered in future improvements of smartphone applications as they need to change over time together with patients changing needs.

An intention-to-treat analysis was conducted even though the rate of self-estimated use of the smartphone application was very different between individuals in the group. We chose this approach because we thought it resembled the ‘use as you wish’ spirit of smartphone applications in general and we wanted to test what would happen if we provided a smartphone application to our patients. The participants in our study were in general satisfied with the rehabilitation and the information after the operation, and there was no difference between the groups. Novak et al.^28^ reported that both video and written instructions during rehabilitation after treatment for mallet fingers were helpful, but those who watched the video instruction perceived the instructions as more helpful than only using the written instruction. The reasons for preferring video or written instruction are different.^28,29^ This makes it important to assess in what way each individual patient wants to receive their instructions during rehabilitation, and as therapists we must be able to provide instructions in that way.

In summary, although our study failed to confirm all the hypotheses, we provide new insights into rehabilitation after flexor tendon injury. Adherence and self-efficacy have not previously been quantitatively explored in this patient group, and previous research suggests promising implementations. The acute nature of flexor tendon injuries also provides different underlying reasons for adherence to rehabilitation exercise compared to more chronic conditions that previously have been the main subjects of adherence research. More attention to adherence is needed in future research in order to better understand its possible link to outcome.

Further research is also needed on which underlying smartphone application features that could make an intervention successful in improving adherence in this patient group. As the design, features and technical possibilities improve in smartphone applications the question remains if a better smartphone application than ours would be able to improve adherence or outcome after flexor tendon repair.

**Clinical message**

- The smartphone application used in this study did not improve adherence, self-efficacy or range of motion compared to standard rehabilitation for flexor tendon injury. Further research regarding smartphone applications is needed.

## Supplemental Material

Appendices – Supplemental material for A smartphone application to facilitate adherence to home-based exercise after flexor tendon repair: A randomised controlled trial Click here for additional data file. Supplemental material, Appendices for A smartphone application to facilitate adherence to home-based exercise after flexor tendon repair: A randomised controlled trial by Jonas Svingen, Jenny Rosengren, Christina Turesson and Marianne Arner in Clinical Rehabilitation

## References

[1] Rigo IZ Røkkum M. Predictors of outcome after primary flexor tendon repair in zone 1, 2 and 3. J Hand Surg Eur Vol 2016; 41(8): 793–801. 27406934 10.1177/1753193416657758

[2] Hitchcock TF Light TR Bunch WH, et al The effect of immediate constrained digital motion on the strength of flexor tendon repairs in chickens. J Hand Surg 1987; 12(4): 590–595. 10.1016/s0363-5023(87)80213-7 3611657

[3] Wada A Kubota H Miyanishi K, et al Comparison of postoperative early active mobilization and immobilization in vivo utilising a four-strand flexor tendon repair. J Hand Surg Br 2001; 26(4): 301–306. 11469830 10.1054/jhsb.2000.0547

[4] Harris SB Harris D Foster AJ, et al The aetiology of acute rupture of flexor tendon repairs in zones 1 and 2 of the fingers during early mobilization. J Hand Surg Br 1999; 24(3): 275–280. 10433435 10.1054/jhsb.1998.0212

[5] Su BW Solomons M Barrow A, et al Device for zone-II flexor tendon repair. A multicenter, randomized, blinded, clinical trial. J Bone Joint Surg Am 2005; 87(5): 923–935. 15866953 10.2106/JBJS.C.01483

[6] Essery R Geraghty AWA Kirby S, et al Predictors of adherence to home-based physical therapies: a systematic review. Disabil Rehabil 2017; 39(6): 519–534. 27097761 10.3109/09638288.2016.1153160

[7] Dobbe JG van Trommel NE Ritt MJ. Patient compliance with a rehabilitation program after flexor tendon repair in zone II of the hand. J Hand Ther 2002; 15(1): 16–21. 11871360 10.1053/hanthe.2002.v15.01516

[8] Cole T Robinson L Romero L, et al Effectiveness of interventions to improve therapy adherence in people with upper limb conditions: a systematic review. J Hand Ther 2017; 32(2): 175–183.E2. 29292028 10.1016/j.jht.2017.11.040

[9] Lambert TE Harvey LA Avdalis C, et al An app with remote support achieves better adherence to home exercise programs than paper handouts in people with musculoskeletal conditions: a randomised trial. J Physiother 2017; 63(3): 161–167. 28662834 10.1016/j.jphys.2017.05.015

[10] Araujo CC Marques AAD Juliato RTC The adherence of home pelvic floor muscles training using a mobile device application for women with urinary incontinence: a randomized controlled trial. Female Pelvic Med Reconstr Surg. Epub ahead of print January 2019. DOI:10.1097/SPV.0000000000000670. 30624250

[11] Brewer WB. Preliminary psychometric evaluation of a measure of adherence to clinic-based sport injury rehabilitation. Phys Ther Sport 2000; 1: 68–74.

[12] Brewer BW Avondoglio JB Cornelius AE, et al Construct validity and interrater agreement of the sport injury rehabilitation adherence scale. J Sport Rehabil 2002; 11(3): 170–178.

[13] Kolt GS Brewer BW Pizzari T, et al The sport injury rehabilitation adherence scale: a reliable scale for use in clinical physiotherapy. Physiotherapy 2007; 93(1): 17–22.

[14] Milne M Craig H Lorie F. Self-efficacy. imagery use, and adherence to rehabilitation by injured athletes. J Sport Rehabil 2005; 14(2): 150–167.

[15] Wesch N Hall C Prapavessis H, et al Self-efficacy, imagery use, and adherence during injury rehabilitation. Scand J Med Sci Sports 2012; 22(5): 695–703. 21496107 10.1111/j.1600-0838.2011.01304.x

[16] Sordoni C Hall C Forwell L. The use of imagery in athletic injury rehabilitation and its relationship to self-efficacy. Physiother Can 2002; 54(3): 177–185.

[17] Jarnbert-Pettersson H Vixner L. Labour Q1 pain - poorly analysed and reported: a systematic review. BMC Pregnancy Childbirth 2018; 18(1): 483. 30526516 10.1186/s12884-018-2089-2 PMC6286546

[18] Park LG Howie-Esquivel J Dracup K. A quantitative systematic review of the efficacy of mobile phone interventions to improve medication adherence. J Adv Nurs 2014; 70(9): 1932–1953. 24689978 10.1111/jan.12400

[19] Bittel CD Bittel JA Williams JC, et al Improving exercise performance with an accelerometer-based smartphone app: a randomized controlled trial. Am J Phys Med Rehabil 2017; 96(5): 307–314. 27610553 10.1097/PHM.0000000000000618

[20] Choi Y Nam J Yang D, et al Effect of smartphone application-supported self-rehabilitation for frozen shoulder: a prospective randomized control study. Clin Rehabil 2018; 33(4): 653–660. 30526016 10.1177/0269215518818866

[21] Wiig ME Dahlin LB Fridén J, et al PXL01 in sodium hyaluronate for improvement of hand recovery after flexor tendon repair surgery: randomized controlled trial. PLoS One 2014; 9(10): e110735. 10.1371/journal.pone.0110735 PMC4207831 25340801

[22] Strickland JW Glogovac SV. Digital function following flexor tendon repair in Zone II: a comparison of immobilization and controlled passive motion techniques. J Hand Surg Am 1980; 5(6): 537–543. 7430595 10.1016/s0363-5023(80)80101-8

[23] Frost R Levati S McClurg D, et al What adherence measures should be used in trials of home-based rehabilitation interventions? A systematic review of the validity, reliability, and acceptability of measures. Arch Phys Med Rehabil 2017; 98(6): 1241–56.e45. 10.1016/j.apmr.2016.08.482 27702555

[24] Lyngcoln A Taylor N Pizzari T, et al The relationship between adherence to hand therapy and short-term outcome after distal radius fracture. J Hand Ther 2005; 18(1): 2–8; quiz 9. 15674780 10.1197/j.jht.2004.10.008

[25] Chen C Neufeld P Feely C, et al Factors influencing compliance with home exercise programs among patients with upper-extremity impairment. Am J Occup Ther 1999; 53(2):171–180. 10200840 10.5014/ajot.53.2.171

[26] World Health Organization. Adherence to long-term therapies: evidence for action. Geneva: Geneva: World Health Organization, 2003.

[27] Huang HP Wen FH Tsai JC, et al Adherence to prescribed exercise time and intensity declines as the exercise program proceeds: findings from women under treatment for breast cancer. Support Care Cancer 2015; 23(7): 2061–2071. 25527243 10.1007/s00520-014-2567-7

[28] Novak CB Mak L Chang M. Evaluation of written and video education tools after mallet finger injury. J Hand Ther 2019; 32(4): 452–456. 30017408 10.1016/j.jht.2018.03.005

[29] Ouegnin A Valdes K. Client preferences and perceptions regarding a written home exercise program or video self-modeling: a cross-sectional study. J Hand Ther 2019; 33(1): 67–72. 30679087 10.1016/j.jht.2018.09.006

