# Supplementary material for: A smartphone application to facilitate adherence to home-based exercise after flexor tendon repair: A randomised controlled trial
Source: Clin Rehabil. 2020 Oct 11;35(2):266–75. doi: 10.1177/0269215520962287 (PMC7874382; doi:10.1177/0269215520962287)
Supplement: Appendices – Supplemental material for A smartphone application to facilitate adherence to home-based exercise after flexor tendon repair: A randomised controlled trial [file Appendices.pdf]

Appendix 1

Sport Injury Rehabilitation Adherence Scale (SIRAS)

Circle the number that best indicates the accuracy with which this patient completed rehabilitation exercises since last appointment?

☐ ☐ ☐ ☐ ☐

Minimum effort — Maximum effort

Since last appointment, how frequently did this patient follow your instructions and advice?

☐ ☐ ☐ ☐ ☐

Never — always

How receptive was this patient to change in the rehabilitation program?

☐ ☐ ☐ ☐ ☐

Very unreceptive — Very receptive

Brewer, W B. Preliminary psychometric evaluation of a measure of adherence to clinic-based sport injury rehabilitation. Physical therapy in sport. 2000;1:68-74.

Appendix 2

Hur många gånger om dagen vill sjukgymnasten/arbetsterapeuten att du ska träna?

- ☐ Vet ej
- ☐ Var fjärde eller färre
- ☐ Var tredje timme
- ☐ Varannan timme
- ☐ Varje timme

Hur många gånger om dagen brukar du faktiskt träna?

- ☐ Vet ej
- ☐ Var fjärde timme eller färre
- ☐ Var tredje timme
- ☐ Varannan timme
- ☐ Varje timme

Med hur många repetitioner vill sjukgymnasten/arbetsterapeuten att du ska träna?

- ☐ Vet ej
- ☐ Ca 3 repetitioner
- ☐ Ca 5 repetitioner
- ☐ Ca 7 repetitioner
- ☐ 10 repetitioner eller mer

Med hur många repetitioner har du faktiskt tränat?

- ☐ Vet ej
- ☐ Ca 3 repetitioner
- ☐ Ca 5 repetitioner
- ☐ Ca 7 repetitioner
- ☐ 10 repetitioner eller mer

Appendix 3

How many times a day did the physiotherapist want you to exercise?

- ☐ Do not know
- ☐ Every fourth hour or less
- ☐ Every third hour
- ☐ Every second hour
- ☐ Every hour

How many times a day have you been exercising?

- ☐ Do not know
- ☐ Every fourth hour or less
- ☐ Every third hour
- ☐ Every second hour
- ☐ Every hour

How many repetitions did the physiotherapist want you to complete during exercise?

- ☐ Do not know
- ☐ About 3 repetitions
- ☐ About 5 repetitions
- ☐ About 7 repetitions
- ☐ 10 repetitions or more

How many repetitions have you been completing?

- ☐ Do not know
- ☐ About 3 repetitions
- ☐ About 5 repetitions
- ☐ About 7 repetitions
- ☐ 10 repetitions or more

Appendix 4

Hur många procent (%) av tiden upplever du att du har tränat korrekt? Markera på linjen med ett kryss. ____

0% 100%

Appendix 5

For what percentage (%) of the time do you experience good quality exercise? Mark the line with a X. ____

0% 100%

Appendix 6

Swedish translation of the Athletic Injury Self-Efficacy Questionnaire.

Skatta på en skala från 0% till 100% i vilken grad du håller med om nedanstående påståenden. Markera med ett kryss på linjen.

Jag är säker på att jag kan..

1. utföra alla övningar i träningsprogrammet

0% 100%

2. följa instruktionerna från min fysioterapeut

0% 100%

3. komma ihåg alla mina övningar

0% 100%

4. genomföra hela mitt träningsprogram även om jag är trött

0% 100%

5. genomföra mitt träningsprogram även om jag känner mig nedstämd

0% 100%

6. genomföra mitt träningsprogram även när jag har ont om tid

0% 100%

7. genomföra mitt träningsprogram även om jag känner viss obehagskänsla

0% 100%

8. genomföra mitt träningsprogram regelbundet oavsett vad som händer

0% 100%

9. följa träningsprogramet enligt det schema som jag fått av min fysioterapeut

0% 100%

10. övervinna alla hinder som kan hindra mig från att utföra mitt träningsprogram regelbundet

0% 100%

Wesch N, Hall C, Prapavessis H, Maddison R, Bassett S, Foley L, et al. Self-efficacy, imagery use, and adherence during injury rehabilitation. Scand J Med Sci Sports. 2012;22(5):695-703.
